# The miniaturized enzyme-modified comet assay for genotoxicity testing of nanomaterials

**DOI:** 10.3389/ftox.2022.986318

**Published:** 2022-10-12

**Authors:** N. El Yamani, E. Rundén-Pran, A. R. Collins, E. M. Longhin, E. Elje, P. Hoet, I. Vinković Vrček, S. H. Doak, V. Fessard, M. Dusinska

**Affiliations:** ^1^ Health Effects Laboratory, Department for Environmental Chemistry, NILU—Norwegian Institute for Air Research, Kjeller, Norway; ^2^ Comet Biotech AS, Department of Nutrition, University of Oslo, Oslo, Norway; ^3^ Laboratory of Toxicology, Unit of Environment and Health, Department of Public Health and Primary Care, KU Leuven, Leuven, Belgium; ^4^ Institute for Medical Research and Occupational Health, Zagreb, Croatia; ^5^ *In Vitro* Toxicology Group, Institute of Life Science, Swansea University Medical School, Swansea, United Kingdom; ^6^ Toxicology of Contaminants Unit, Fougères Laboratory, French Agency for Food, Environmental and Occupational Health and Safety, Fougères, France

**Keywords:** alkaline comet assay, nanomaterial, genotoxicity, DNA damage, interference, lesion-specific endonucleases, oxidized DNA bases, 12 mini-gels

## Abstract

The *in vitro* comet assay is a widely applied method for investigating genotoxicity of chemicals including engineered nanomaterials (NMs). A big challenge in hazard assessment of NMs is possible interference between the NMs and reagents or read-out of the test assay, leading to a risk of biased results. Here, we describe both the standard alkaline version of the *in vitro* comet assay with 12 mini-gels per slide for detection of DNA strand breaks and the enzyme-modified version that allows detection of oxidized DNA bases by applying lesion-specific endonucleases (e.g., formamidopyrimidine DNA glycosylase or endonuclease III). We highlight critical points that need to be taken into consideration when assessing the genotoxicity of NMs, as well as basic methodological considerations, such as the importance of carrying out physicochemical characterization of the NMs and investigating uptake and cytotoxicity. Also, experimental design—including treatment conditions, cell number, cell culture, format and volume of medium on the plate—is crucial and can have an impact on the results, especially when testing NMs. Toxicity of NMs depends upon physicochemical properties that change depending on the environment. To facilitate testing of numerous NMs with distinct modifications, the higher throughput miniaturized version of the comet assay is essential.

## Graphical Abstract

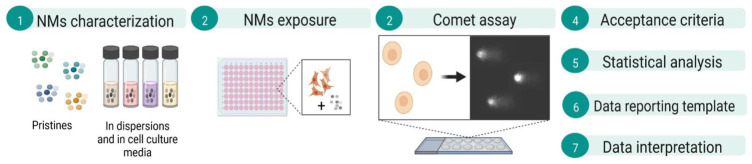

## 1 Introduction

A recent paper in the journal Nature Protocols describes in detail the various protocols for the *in vitro* comet assay (Collins et al., 2022). Here we focus on the *in vitro* testing of nanomaterials (NMs) using the alkaline comet assay based on 12 mini-gels per slide, in combination with lesion-specific endonucleases.

We address the most relevant points to be taken into consideration when assessing NM genotoxicity. Hazard assessment of NMs with conventional methods for chemical testing poses a challenge, owing to physicochemical properties of NMs, such as optical features, reactivity, and surface area, which differ from those of the corresponding bulky chemicals. NMs may interfere with the test assay endpoints, especially those relying on colorimetry or fluorimetry principles, leading to potentially biased data (Guadagnini et al., 2015; Karlsson et al., 2015).

The particular physicochemical properties of NMs may lead to potential interference with standard test methods including the comet assay. Certain NMs, such as TiO_2_, and nanogold, are especially likely to cause interference. The possibility of interference with the comet assay by NMs has been discussed previously (Kain et al., 2012; Magdolenova et al., 2012; Karlsson et al., 2015; Di Bucchianico et al., 2017; George et al., 2017). Interference may happen either directly or indirectly: 1) direct/physical interference of the NMs with the DNA (after lysis) creating additional breaks or adducts; 2) possibilities for NMs to interfere by reducing or blocking the DNA migration during electrophoresis; 3) inhibition/interaction with Fpg activity; 4) quenching/autofluorescence during quantification of signals/scoring; 5) interference of photosensitive particles with direct light may cause changes in the particles (e.g., increase their reactivity or effect). We therefore suggest here that proper interference controls should always be included in the experimental design.

The effect of DNA damaging agents can be detected by a wide range of toxicology assays. The single cell gel electrophoresis (or comet assay), is widely used for detection of DNA damage induced by chemicals, and is the most used method for testing NMs (Magdolenova et al., 2012; Huk et al., 2015a; Magdolenova et al., 2015; El Yamani et al., 2017; Garcia-Rodriguez et al., 2019). The alkaline comet assay measures DNA damage (single and double strand breaks and alkali-labile sides) in eucaryotic cells (Collins, 2004; Collins et al., 2017a; Collins et al., 2017b; Gajski et al., 2019; Collins et al., 2022). Since it was introduced in 1984 (Ostling and Johanson, 1984), the assay has gone through several modifications to increase sensitivity and reduce variability, as well as to increase its robustness and applicability in different areas. While the *in vivo* comet assay has been validated, and OECD Test Guideline (TG 489) approved (OECD, 2014b), there is not yet any OECD test guideline for the *in vitro* comet assay. The protocol for testing NMs by the *in vivo* comet assay is described by Elsepuru et al. (2022).

The *in vitro* comet assay has been miniaturized to allow many more samples to be analysed in a single experiment. Thus, 12 mini-gels are applied to one slide instead of the one or two gels as in the original procedure; or 96 mini-gels can be placed on a GelBond film (Azqueta et al., 2013; Gutzkow et al., 2013). A commercial ‘microarray’ assay (CometChip) has also been developed (Watson et al., 2014). Scoring of comets in gels on the slides is time-consuming, and this presents a bottle neck in the performance of the assay, although semi-automated image analysis systems are available (Dusinska and Collins, 2008; Collins et al., 2022). Automated image analysis systems are also available (e.g., Metafer from Metasystems, Germany). To increase its sensitivity and to detect diverse types of lesions, the assay has been modified by the inclusion of a digestion with lesion-specific enzymes after the lysis step; thus otherwise undetectable base damage is converted into abasic sites and single strand breaks (SBs) are introduced (Dušinská and Collins, 1996; Olive, 2002). The most used enzymes are endonucleases specific for DNA base oxidation, namely formamidopyrimidine DNA glycosylase (Fpg) (Dušinská and Collins, 1996) or the mammalian counterpart, 8-oxoguanine DNA glycosylase (OGG1) which cleave oxidized purines, and endonuclease III (Endo III) for oxidized pyrimidines (Collins et al., 2014; Collins, 2017).

In this manuscript, we focus on application of the *in vitro* 12 mini-gel format alkaline version of the comet assay in combination with lesion-specific endonucleases (e.g., Fpg or Endo III) for detection of both DNA SBs and oxidized DNA bases induced by NMs. We are addressing the most relevant points that need to be taken into consideration when assessing NM genotoxicity. Interpretation of NM comet assay data is facilitated by a categorization approach for positive, negative and equivocal effects recently developed within the H2020 NanoREG2 project (El Yamani et al., 2022).

A thorough physicochemical characterization of the NMs, both pristine as well as in culture medium should be always provided before toxicity testing. When performing genotoxicity, the cytotoxicity of the NMs to identify concentration range and the highest concentration must be conducted adequately. Last but not least, cellular uptake should be also investigated.

## 2. Materials and equipment

### 2.1 Materials

#### 2.1.1 Consumables and reagents

Cells (adherent or suspension cells), Flasks 25 cm^2^ or/and 75 cm^2^, Glass microscopic slides, Cover slips 22 mm × 22 mm or 22 mm × 60 mm, Sterile plastic centrifuge tubes 15 ml and 50, Pasteur pipettes 2, 5 and 10 ml, 96-well plates, Microcentrifuge tubes (1.5, 5 ml), Serological pipettes, Pipette tips.

Cell culture medium (according to cell line) and additives (serum, penicillin-streptomycin, etc.), trypsin-EDTA solution (CAS. 59429C, Sigma), phosphate buffered saline (PBS) (Thermo Fisher, 10010049), dimethyl sulfoxide (DMSO) (Sigma-Aldrich, cat. number D5879- CAS. 67-68-5), Trypan Blue stain (Thermo Fisher, cat number 15250), Agarose—Electrophoresis grade normal melting point (NMP) (Fluka, cat number 05066), Agarose Low melting point (LMP) (Sigma-Aldrich, cat number A9414), distilled water, ethanol, Triton X-100 (Sigma-Aldrich, cat number T8787), Bovine serum albumin (BSA) (Sigma-Aldrich, cat number A9418), CaCl_2_ (Mw = 74.55), MgCl_2_, H_2_O_2,_ 30%; (Sigma-Aldrich, cat number 31642-M), NaOH, Na_2_EDTA (CAS 6381-92-6 SIGMA), Tris base (CAS 77-86-1 CALBICHEM), NaCl (CAS 7647-14-5 SIGMA), KrBO_3_ (CAS 7758-01-2), KCl (CAS 7447-40-7 Sigma), HEPES (CAS7365-45-9 Sigma), KOH (Mw = 56.11), methymethane sulphonate (MMS) (CAS. M4016_ Sigma Aldrich), Fpg, Endo III, SYBR^®^ Gold (Thermo Fisher S11494) (or other stains such as DAPI (4′,6-diamidino-2-phenylindole),PI (propidium idiode).

#### 2.1.2 Equipment and software

Laminar flow hood, light microscope, countess cell counter or Bürker chamber with cover glass, pipettes, automatic pipettes and multi channel pipette (optional), microwave oven, CO_2_ incubator, centrifuge, water bath or heat block, fridge 4°C, Incubator 37°C, electrophoresis equipment with power supplier, fluorescent microscope (with CCD camera).

For scoring comets, the use of a computer-assisted image analysis system with commercially available software is recommended to give the most reproducible results. Examples of scoring softwares: Comet assay IV (Instem), Comet Analysis software (Trevigen), Lucia Comet Assay™ software (Laboratory Imaging), Metafer (MetaSystems), KOMET 6 (Andor Technology). Several free scoring programs are also available such as Casplab or CometScore. The visual scoring system is an alternative (Dusinska and Collins, 2008) and (Collins et al., 2022).

#### 2.1.3 Preparation of slides and solutions

##### 2.1.3.1 Pre-coating of microscopic glass slides

Ordinary grease-free microscopic glass slides are pre-coated with (0.5%) normal melting point (NMP) agarose. To prepare 100 ml of agarose solution, weigh 0.5 g NMP agarose and dissolve in 99.5 ml distilled H_2_O by heating in a microwave oven. Fill a suitable vessel (Coplin jar or a narrow beaker) with the hot NMP agarose solution and place it in a water bath or a heat block set at (55**°**C) for approximately 15 min before using it as described below step by step:

• Dip one clean microscope slide vertically in the solution of agarose by holding it from the frosted area.
• Drain off excess agarose by holding the slide vertically for some seconds, then wipe the back of the slide with a tissue and leave the slide horizontally on the bench to dry overnight.
• Mark the coated side with a pencil mark in one corner on the frosted end (e.g., top left) to identify the coated side.
• Dried pre-coated slides can be stacked together in slide boxes and stored at room temperature for several months.

Note. Commercially precoated slides are also available and can be purchased.

##### 2.1.3.2 Preparation of low melting point agarose solution

The LMP agarose solution is made in PBS. The concentration can vary between 0.6 and 1% depending on the cell type and genome complexity. For instance, a lower percentage % of LMP agarose can be recommended when working with plants. For cultured cells, we recommend 0.8% LMP agarose. The agarose can be prepared in batches and stored at 4°C in a fridge. LMP agarose is dissolved in PBS by careful heating in a microwave oven; after about 10-15 s, shake the flask to ensure uniform heating; repeat until the fluid is clear and the agarose completely dissolved. Make small aliquots (e.g.,10 ml per bottle/falcon tube) and keep at 4°C.

##### 2.1.3.3 Lysis solution

The preparation of lysis takes several hours to dissolve all reagents and to adjust pH. The lysis solution (2.5 M NaCl, 0.1 M Na_2_EDTA, and 0.01 M Tris-base) is therefore usually prepared ahead in distilled H_2_O and kept at 4°C. Generally, all the ingredients are weighed and added before adding distilled H_2_O. Triton X-100 at 1% is added to the lysis solution before use. The solution should be mixed properly using magnetic blender and kept at 4°C until use.

##### 2.1.3.4 Enzyme reaction buffer for Fpg

The enzyme reaction buffer (0.04 M HEPES, 0.10 M KCl, 0.0005 M EDTA, 0.2 mg/ml BSA) is prepared in H_2_O and the pH is adjusted to 8.0 using KOH (e.g., 8 M). The buffer is used to dilute the enzyme to the desired concentration. The buffer can be used for both Fpg and Endo III.

##### 2.1.3.5 Electrophoresis solution

The electrophoresis solution (0.3 M NaOH, 0.001 M Na_2_EDTA) is prepared in distilled H_2_O and kept at 4°C.

##### 2.1.3.6 TRIS-EDTA for dilution of SYBR®Gold

The TRIS-EDTA (TE) buffer (2.5 mM Tris-base, 4 mM Na_2_EDTA) is prepared in distilled H_2_O and pH adjusted to pH 7.5–7.8 (e.g., HCl). The buffer is also commercially available.

##### 2.1.3.7 SYBR®Gold solution

To avoid repeated thawing and freezing, the commercially purchased SYBR®Gold stock can be aliquoted after first thawing (e.g., 50 μl in microtubes) and stored at −20°C. The SYBR®Gold may be further diluted in DMSO and stored at −20°C. On the day of staining of the slides, the stock dye is diluted 10 times in TE buffer. For the 12 mini-gels staining, a drop of diluted SYBR®Gold (20 μl) is placed on top of each gel. The slide is covered with coverslip 22 mm × 60 mm and placed in dark for 5–10 min before visualization under fluorescence microscope.

Be aware DNA dyes are carcinogenic and should be handled with care. Use gloves and collect the waste in a hazard-labelled container.

##### 2.1.3.8 Enzyme preparation

The lesion specific enzymes used in combination with the comet assay are commercially available from different sources. The purchased enzymes are usually followed with instructions for their use. Here, we are describing the procedure for two lesion-specific enzymes used to detected oxidized bases, Fpg and Endo III. These enzymes are isolated from bacteria containing over-producing plasmids. Upon receipt, they should be dispensed into small aliquots (e.g., 5 μl) and stored at −80°C. The final dilution of the working solution varies from batch to batch. A titration of the enzyme is used to find the optimum dilution for comet experiments and is usually carried out by the supplier. The stock solution is diluted using the Fpg reaction buffer described above, with the addition of 10% glycerol; aliquots are stored at −80°C. For use in an experiment, the Fpg is thawed and further diluted with Fpg buffer (no glycerol is needed) following instructions from supplier. It is usually recommended to keep the aliquots all the time on ice until adding to the gels. If any of this working solution is left over, do not refreeze.

## 3 Methods

**FIGURE 1:**
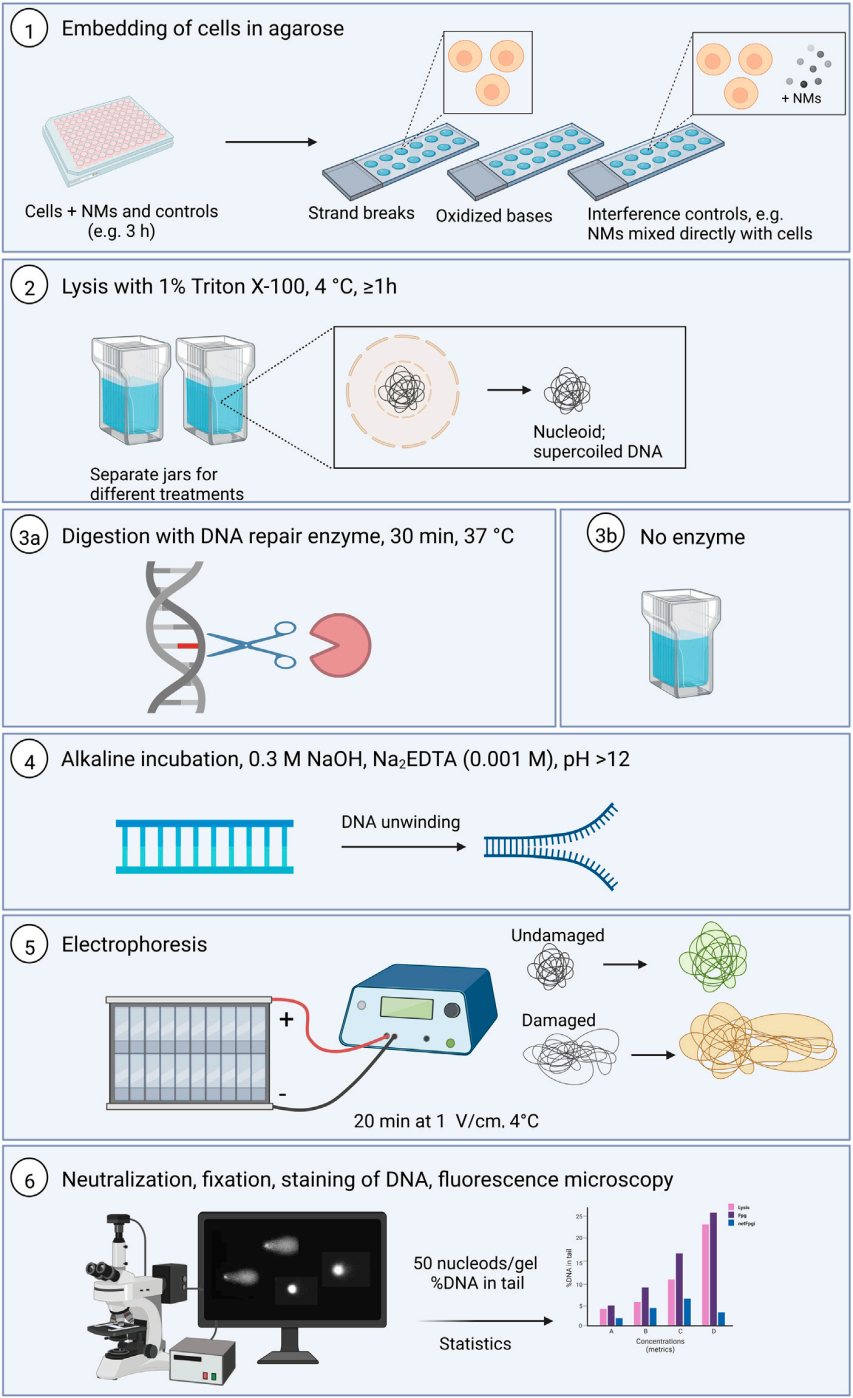
Summary of the comet assay protocol for both standard and the enzyme-modified version (Created with Biorender.com). 1. Cells are seeded in correct density using 96 well format and exposed to the NMs and controls and after exposure time, the cells are embedded with 0.8% LMP agarose to make 12-gel format slides. 2. Lysis incubation at 4°C for at least 1 h 3a. the slide with samples to be incubated with DNA repair enzyme to reveal oxidative damage are incubated with the enzyme for 30 min at 37°C. 3b. The slides with samples for DNA strand breaks detection remain in the lysis solution. 4. All the slides are placed in the alkaline solution for DNA unwinding. 5. Electrophoresis is run for 20 min at 1V/cm. 6. At the end of the unwinding, all the slides are washed by the neutralization solution, fixed and then stained before visualization and scoring. 50 nucleoids are analyzed per sample or gel. %DNA in tail parameter is collected and statistical analyses performed. h, hours; d, days; LMP, low melting point.

The standard alkaline comet assay procedure has been described in various papers, and in exhaustive detail in a recent Nature Protocols paper (Collins et al., 2022). Here we emphasize the particular considerations that need to be taken into account when applying it to NMs, but a brief outline of the overall procedure is in order. The principle of the assay is that strand breaks release the supercoiling in DNA loops and allow the DNA to extend towards the anode under electrophoresis, forming comet-like structures; the proportion of DNA in the tail represents the frequency of DNA breaks. A summary of the comet steps is presented in the Figure 1 below.

Cells that have been experimentally exposed to a NMs, accompanied by appropriate control cells, are mixed with LMP agarose and set as gels on a microscope slide (two large gels or 12 mini-gels) or on a GelBond film (up to 96 gels in a 12 × 8 array) or in more elaborated devices such as CometChip. The cells are lysed with high salt and detergent, leaving the DNA attached to the nuclear matrix as a so-called nucleoid. Digestion with lesion-specific endonuclease is an option at this stage. Electrophoresis follows, and the comets (typically 100 per sample) are quantitated using image analysis software or by visual scoring (Dusinska and Collins, 2008; Collins et al., 2022).

Due to its high sensitivity and to ensure reproducibility and reduce variability in the results, it is recommended to perform comet assay experiments always in the same manner following a standardized approach and experimental design taking into consideration, amount of medium to be used per treatment, plate layout type, dispersion of NMs and the series of controls (including agent control and reference standards) to be included (Dusinska et al., 2019). Moreover, historical data for negative (NC) and positive (PC) controls should be stored as they are key information for conclusion statement.

Additionally, to NC, capping agents’ control, PC and interference controls, at least 4 concentrations of the test substance should be included.

The length of exposure to NMs is also crucial to consider as it should be sufficient for damage to occur. The comet assay normally measures an acute response and thus for testing chemicals *in vitro* an exposure time from 5 min (e.g., H_2_O_2_) to 24 h is usually recommended (Dusinska et al., 2019). However, for NMs testing we recommend at least three hours to ensure cellular uptake. An access to DNA could be dependent on dissolution of the nuclear membrane during mitosis (Catalán et al., 2014). Partly soluble NMs could exert their effects in shorter time. Generally, we advise both short (e.g., 3 h) and long (e.g., 24 h) exposure to be conducted within the same experiment. Three independent experiments (including at least two duplicates) are recommended.

When preparing the slides with 12 mini-gels and to increase the robustness of the results, it is recommended to include also replicate gels and replica slides in each experiment. Based on our experience, and due to the high sensitivity of this assay, it is also advised that the PC treatment should be placed in a separate plate or at least with empty wells separating them from the other samples. Also, when preparing gels on slides, the gels with cells treated with the PC should be made in separate slides.

### 3.1 Cell lines and preparation of culture

The comet assay has the advantage that it can be performed in both proliferating and non-proliferating cells. Any cell type with a nucleus can be used, and thus the assay can assess both cell- and tissue-specific DNA damage induced by NMs (Dusinska et al., 2019; Collins et al., 2022). For *in vitro* genotoxicity testing and for human hazard assessment of NMs, human and other mammalian cells such as from lung (e.g., A549 and Beas-2B cells), liver (e.g., HepG2 cells), circulatory system (e.g., THP1 or TK6 cells) are commonly used. The cells should be viable, and preferably at low passage (P). A guidance document was recently published about best practices in all aspects of the *in vitro* use of cells and tissues (Pamies et al., 2022). In this method paper, an example of adherent cells is given using the lung A549 cells. These cells (ECACC) grow in DMEM D6046 (low glucose with 4 mM L-glutamine) (Sigma), 9% fetal bovine serum (FBS) (26140-079, ThermoFisher), 100 U/ml penicillin/100 μg/ml streptomycin solution (15140-122, ThermoFisher). Suspension cells, such as human lymphoblastoid TK6 cells (ECACC) are grown in RPMI 1640 without glutamine (31870, GIBCO^®^, Life Technologies), 9% Horse Serum (16050122, GIBCO^®^, Life Technologies or H1138, Invitrogen), L-Glutamine 200 mM (25030-024, GIBCO^®^, Life Technologies), 100 U/ml penicillin/100 μg/ml streptomycin solution (15140-122, ThermoFisher). Cells are grown in complete culture medium and incubated in culture dishes or flasks in a cell incubator with humidified atmosphere at 37°C, 5% of CO_2_ following the standard operating procedure (SOP) for cultivation of the specific cell line.

### 3.2 Seeding of cells for exposure

The seeding of cells can be conducted in any type of plate layout. However, to increase the throughput and the robustness of this assay, the use of 96 well plate format for cell seeding is recommended. The number of cells per well is dependent on the cell type and doubling time. For instance, for A549 cells, with a doubling time of about 22 h, it is recommended to seed cells 24 h before exposure to reach adequate confluency before exposure (70%–80%). A549 cells are normally seeded between 10.000–15.000 cells/well in 200 μl of medium in duplicate in a 96 well plate format. For TK6 cells, the seeding can be conducted on the same day or the day before exposure since the cells are in suspension. The cells are seeded at 15.000–20.000 cells/well in a 96 well plate in 200 μl final volume of medium.

### 3.3 Preparation of controls and nanoparticles

#### 3.3.1 Negative controls

Concurrent NC handled in the same way as the treatment cultures should be included for every experimental condition as recommended by ENV/JM/MONO(2016) (OECD, 2017). The NC usually consists of cells incubated in the same culture medium for the specific cell line as exposed cells. It can also be the vehicle used such as PBS or DMSO. The vehicle controls should not produce toxic effects and should not be suspected to cause chemical reaction with the test substance.

PBS (with CaCl_2_ and MgCl_2_) may be used as NC but only for exposure times up to 2 h. If DMSO is used as a solvent for the NMs, it should be added to the culture medium or PBS in the same concentration as for the group exposed to the highest concentration of the test substance. The final concentration of DMSO should not exceed 2% (OECD, 2016).

#### 3.3.2 Capping agent controls

The capping agent control(s) which are usually used to prepare the NMs are of utmost importance as stabilizers that inhibit the over-growth of nanoparticles and prevent their aggregation/coagulation in colloidal synthesis (Javed et al., 2020). The quality and the type of the capping agents are responsible for changing NMs physicochemical properties, and the biological characteristics affect they may have. Capping agents should be non-toxic and therefore, investigating their toxic potential separately is important along with testing the NMs suspension. Different types of capping agents have been used in nanoparticles’ synthesis including surfactants, small ligands, polymers, dendrimers, cyclodextrins, and polysaccharides. The Polyethylene glycol (PEG), Polyvinylpyrrolidone (PVP), polyvinyl alcohol (PVA), bovine serum albumin (BSA), ethylene diamine tetra acetic acid (EDTA) and chitosan are the most used capping agents for NMs. Capping agents can also be from plant extracts. Several studies have demonstrated the toxic effect of the NMs capping agents used when tested alone (Huk et al., 2015b). Information on the type of solvent, composition and concentration used need to be provided along with information about the NMs as pristine. The concentration of the capping agent to be tested has to be exactly the same in each cellular sample as in the vehicle control. Test at least a concentration of the capping agent used for the stock solution of the test substance equal to the amount in the highest concentration of the tested NM in the experiment. It is recommended to test also lower concentrations of the capping agents and to establish a concentration response curve.

#### 3.3.3 Positive controls

Concurrent PCs should always be included, to demonstrate the ability of the method to detect a genotoxic effect under the conditions of the test protocol. If the treatment time for the PC is different from the exposure time for the tested NMs, the PC should be added towards the end of the exposure for the NMs so that NMs and PC exposures end at the same time. PCs can be selected according to the criteria of the specific study, the material tested, the method used and whether a metabolic activation system is present/needed. Some PCs may be used as reference standards that are applicable to several methods. In the case of the comet assay, MMS (alkylating agent) and H_2_O_2_ are commonly applied as reference standards and PCs for assay of DNA strand breaks. For DNA oxidized purines (Fpg-sensitive sites), potassium bromate (KBrO_3_), or the photosensitizer Ro19-8022 in combination with visible light are used (see Table 1 for more information). When using H_2_O_2_, it is recommended to treat the cells after embedding for 5–10 min treatment (20–100 μM, 4°C) since with longer incubation H_2_O_2_ loses its activity and alsoDNA breaks are quickly repaired, (Collins et al., 2017a; Collins et al., 2022). The concentration of the PC to be used should be selected so as to produce moderate effects that critically assess the performance and sensitivity of the assay and could be based on concentration response curves established by the laboratory.

**TABLE 1:** Examples of positive control chemicals to be used for the comet assay.

Substance name	Solvent	Diluted further in	Recommended stock concentration	Working concentration	Exposure time	Positive control for
MMS	DMSO + PBS	Cell culture medium	1 mM	0.1–0.3 mM	15–30 min, 1–24 h	DNA strand breaks
H_2_O_2_	—	Cell culture medium	100 mM	50–100 μM	5–30 min	DNA strand breaks
RO19-8022	70% ethanol + PBS	Cell culture medium	1 mM	1–2 μM	4–8 min	DNA oxidisd bases
KBrO_3_	PBS	Cell culture medium	6 M	1–2 mM	3–24 h	DNA oxidisd bases

#### 3.3.4 Preparation of nanomaterials, selection of concentration range and exposure

When testing NMs, proper dispersion of the material needs to be ensured. Information on dispersibility in terms of the relative amount of the NMs that can be dispersed in a suspending medium, including information on stability of the dispersion in the culture medium and the conditions applied should be provided (SCCS, 2019; EFSA et al., 2021). Depending on whether the material is in powder or suspension form, steps such as dispersion and sonication may be required. There is no universally applicable protocol for preparing stable dispersions of NMs, but specific methods for certain types of particles have been published, such as NANOGENOTOX protocol (NANOGENOTOX, 2012) for NM dispersion validated under several EU projects, namely FP7 NANoREG and H2020 NanoREG2 as well as H2020 PATROLS and RiskGONE. The EU-project NanoDefine has developed dispersion protocols for a number of NMs (Mech et al., 2020). The protocol developed by DeLoid et al. (2017) has also been applied by several EU projects, among them H2020 RiskGONE.

When exposing cells to NMs, two concentration metrics are normally considered, either mass per area (μg/cm^2^) or mass per volume (μg/ml). The relationship between both metric units varies depending on the set-up (flask, dish, or multiwell). Other metrics include number of particles per ml or cm^2^ as well as particle surface area per ml or cm^2^. Whatever the concentration metric considered, it is important to provide all the information required to move from one metric to the others so that comparison of data will be facilitated.

The concentrations used for genotoxicity studies should be realistic and relevant to potential human exposure. The concentration range should be established with regard to expected cytotoxicity, solubility in the test system and changes in pH or osmolality (OECD, 2017). At higher concentrations, NMs have a tendency to sediment and to agglomerate, and therefore the highest concentration of NMs in tests should not exceed the level at which agglomeration is initiated (Catalán et al., 2014). The agglomeration of nanoparticles may affect their bioavailability to the cell and thus might lead to false positive/negative results. Within FP7 NanoTEST and NANoREG projects, it was agreed that the highest concentration should generally be less than 100 μg/ml. According to OECD TGs, the highest concentration should be below or up to the first concentration giving precipitation.

In general, at least four concentrations of the tested NMs should be included, plus negative/vehicle control (NC), PC and capping agents. For the comet assay, if the compound is cytotoxic, at least one cytotoxic concentration giving no more than 30% cytotoxicity, and a minimum of three non-cytotoxic concentrations, should be tested.

### 3.4 Nanomaterials primary and secondary characterization

Characterization of the NMs to be tested is a key to understanding their observed effects and their mechanism of action. The physicochemical properties of NMs have been linked to their effect or toxicity in several studies (Huk et al., 2015b; Magdolenova et al., 2015; El Yamani et al., 2022). It is, therefore, important to perform full characterization of the pristine NMs where intrinsic properties will be measured; this is what we refer to as primary characterization. Behavior of NMs depends also on extrinsic properties which can be measured through so-called secondary characterization in the cell culture medium (size distribution, polydispersity, zeta potential, solubility, aggregation/agglomeration). There are several methods/techniques for NMs characterization, the most used being Dynamic Light Scattering (DLS) or NM tracking analysis (NTA) for size distribution and zeta-potential.

### 3.5 Cytotoxicity assessment as part of genotoxicity testing

It should be mentioned that a positive finding with the comet assay may not be due to genotoxicity but may also represent an indirect effect of general cellular toxicity. Therefore, cytotoxicity testing using an appropriate test should always be performed as part of all genotoxicity testing strategies. For the comet assay it is important to distinguish true exogenous DNA damage from the low level of DNA damage in the earliest stage of apoptosis. It has been recommended to limit testing to non-cytotoxic concentrations but a consensus about threshold has not been reached (Azqueta et al., 2022). There are several assays for cytotoxicity, some of which are time consuming when performing high throughput (HTP) analysis. The most used ones are based on colorimetric methods such as AlamarBlue, MTS, MTT, WST-1. However, potential interference of NMs with these methods needs to be tested (see Longhin et al., 2022 this collection of manuscripts). Cytotoxicity should always be tested with the same cells and the same set-up as for the comet assay—plate layout and amount of medium, NMs dispersion etc.—and ideally performed in the same experiment as the comet assay (El Yamani et al., 2017).

### 3.6 Cellular uptake and localisation of nanoparticles

It is now highly recommended to check internalization of NMs in the cells when testing genotoxicity. Accompanying genotoxicity testing with uptake studies is now required by several risk assessment committees (SCCS, 2019; EFSA et al., 2021). The aim is to demonstrate that cells are actually exposed, and that NMs are in contact with cellular organelles and molecules, including DNA. The DNA may be exposed to the NMs also during cell division, and so absence of nuclear uptake does not mean that NMs are not in contact with DNA. Demonstration of cellular uptake is particularly important when negative results are obtained. If such exposure cannot be demonstrated, a negative outcome of the assay might be meaningless, as the target exposure will not be known. However, a positive outcome from a genotoxicity test is not strictly dependent on uptake by the cells as genotoxicity may be induced *via* indirect mechanisms, such as through extracellular stimulation coupled to activation of intracellular signaling cascades, or *via* secondary genotoxicity by extracellular reactive oxygen and nitrogen species (Magdolenova et al., 2014; Dusinska et al., 2017a; Dusinska et al., 2017b). There are several methods to study uptake; most common are electron microscopy, confocal microscopy, Raman spectroscopy, flow cytometry and mass spectrometry (Dusinska et al., 2017b). Among them, transmission electron microscopy (TEM), is the most used (Huk et al., 2015b; Rubio et al., 2016; Kazimirova et al., 2019).

### 3.7 Comet assay procedure

On the day of exposure, the cells seeded in duplicate are exposed to the selected concentrations of the NM including positive(s), capping agents (s) and negative/vehicle controls and placed at 37°C, 5% CO_2_, for the required time. Before the end of the exposure, the lysis solution is mixed with 1% Triton-X as described above. The final lysis solution is kept at 4°C until use. The LMP agarose is carefully heated in the microwave oven until completely melted and placed in a pre-warmed bath or thermo-block at 37°C until use. *Note*. Make sure the LMP agarose is at the right temperature 37°C before mixing it with the cells. Precoated slides should be labeled accordingly following a template.

#### 3.7.1 Embedding of nanomaterials in low melting point agarose

At the end of exposure (day 1 or 2 depending on length of exposure), cells are mixed with LMP agarose. In the 12-gel fomat, each gel of 5–10 μl contains between 200–500 cells which is appropriate for image analysis. The volume of the cell suspension added to agarose to make the slides should not reduce the percentage of agarose to less than 0.45% (see also OECD TG 489 comet *in vivo*). The cell embeding should be done as soon as possible after cell treatment. From each treatment, 1- 2 gels are made on a pre-coated slide, preferably on 2 replicate slides. The slides for NMs interference control are prepared in parallel and as described above with one exception, no incubation time is needed for the NMs to be tested for interference. At the end of exposure, cells from NC and PC are kept to be used for the interference controls. The cells are mixed directly with the tested NMs in a way to achieve the highest tested NM concentration in the mixture. The mixture is then directly embedded in agarose as described above. Interference control slides are then placed into lysis solution and incubated into electrophoresis solution for DNA unwinding before electrophoresis, neutralization etc. as described above. The approach of using cells from the NC and then mixing directly with the NMs has been previously published (Magdolenova et al., 2012). There are also several other approaches to check for NM interference. For instance, for photosensitive NMs, such as TiO_2_ NMs, comparison between results from performing the NMs exposure and embedding of cells on gels under normal light and red light or switching off the light has been suggested (Karlsson et al., 2015).

#### 3.7.2 Immersion of slides in lysis solution

Lysis step is an important step and keeping constant lysis conditions will help avoid variability within experiments. Once prepared, the slides are immersed in cold lysis solution already prepared with 1% Triton-X and at 4°C and incubated for at least 1 h or overnight.

#### 3.7.3 Unwinding in alkaline solution and electrophoresis

At the end of lysis incubation and enzyme incubation, the slides are then placed in the electrophoresis tank filled with cold alkaline electrophoresis solution, side by side, for the unwinding step. This step is also critical and the solution conditions, (e.g., pH, temperature), length of incubation and volume used should be kept constant. The slides (with gel drops) should be totally covered (0.5 cm of solution above). If there are gaps in the tank (few slides), it is recommended to fill the gaps on the platform with some empty slides to maintain the depth of solution over the platform. The period of incubation is usually 20 min at 4°C in dark. At the end of the unwinding step, the electrophoresis is conducted. Electrophoresis should be run at 4°C in a cold room or a fridge if possible. Within the hComet project, conducting electrophoresis at 1 V/cm for 20 min was recommended. The duration of electrophoresis is considered a critical variable and the electrophoresis time should be set to optimize the dynamic range. Longer electrophoresis times (e.g., 30 or 40 min to maximize sensitivity) usually lead to stronger positive responses with certain mutagens. It may also lead to excessive migration in control samples (Collins et al., 2022).

#### 3.7.4 Neutralisation and fixation

After electrophoresis, slides are washd twice in cold PBS for 5 min follwoed by dH_2_O for 5 min. The slide are left to dry horizontally at room temperature (normally overnight). Fixation using 70% ethanol for 15 min followed by absolute ethanol for another 15 min is recommended when using the 12-gel format. The slides are dried overnight and can be stored for months at room temperature as long as they are protected from light and dust.

#### 3.7.5 Staining, image analysis and data collection

Before image analysis, the gels are stained with SYBR®Gold (0.1 μl/ml in TE buffer) or another specific dye such as DAPI (1 μg/ml DAPI solution in distilled H_2_O). Slides are analysed using fluorescence microscopy with a computer image analysis program, e.g., Comet assay IV (Perceptive instruments), Metafer (Metasystems) or by visual scoring. We generally analyse at least 50 comets per gel (2 gels per treatment group). Cells close to the edge of the gel are not scored so as to avoid any potential “edge effects”. It is recommended that every gel is scored “blind” to its treatment. This is the standard practice for studies conducted in a regulatory environment, for example under Good Laboratory Practice (Bright et al., 2011). The % DNA in tail is considered the most informative parameter (Collins, 2004; Møller et al., 2014; Møller et al., 2020; Collins et al., 2022).

**FIGURE 2:**
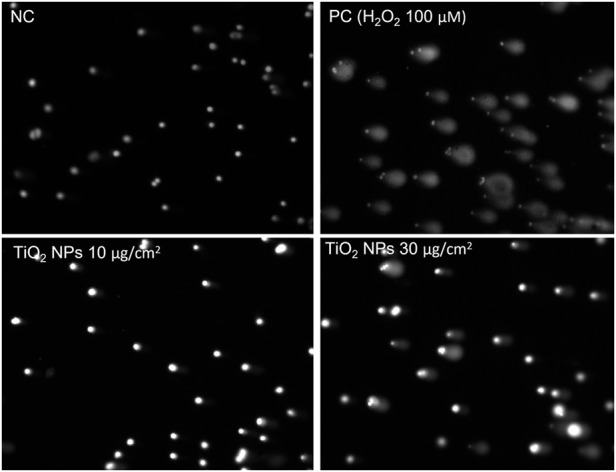
Example of analyzed samples with and without DNA damage. The nucleoids from the unexposed A549 cells (NC) were not affected with the electrophoresis and no increase in the tail was observed. While the nucleoids obtained from cells exposed to the PC (100 μM H_2_O_2_) and to TiO_2_ NMs at 10 and 30 μg/cm^2^ for 3 h show an increase in DNA migrated outside the nucleoids head forming a tail. The image analysis was performed after staining of the samples with SyberGold. For the image analysis Comet IV (Perceptive instruments) was used at ×100 magnification.

An example of results from non treated A549 cells (NC) and cells treated with TiO_2_ nanoparticles are shown in Figure 2.

At the end of image analysis, the data are collected in suitable templates. To facilitate data collection and interpretation, the use of harmonized templates to report comet data is highly recommended. Within several EU projects, data collection template has been introduced for the comet assay, i.e., by the FP7 NanoTEST and NANoREG projects. The template has been further improved within H2020 RiskGONE to move towards data reporting, harmonization, and data FAIRness. The template provides a function for automatic calculation of the results from the reported raw data. The template is available upon request through the eNanomapper database (www.enanomapper.com), and it will be made publicly available. In Figure 1, we summarized the main steps of this assay.

## 4 Data analysis and statistics

The use of an appropriate statistical programme is recommended (e.g., Excel, GraphPad Prism, SPSS). In general, data from comet assay are processed as follows:

• Calculate the median of the ±50 comets (% DNA in tail) per gel replicate.
• Calculate the mean of medians and standard deviation SD (for the replicate gels of the same concentration/sample within the same experiment). Then, calculate the mean value ±SD for all independent experiments (at least three independent experiments are recommended).
• Compare the DNA damage of the PC with NC (control group),
• Compare the DNA damage of the tested NMs with NCcontrol group). Consider differences between replicates, differences between controls and treated cells, correlations and concentration response relationship.

The choice of the statistical tests to be applied, parametric or non-parametric tests, depends on many factors such as size of the data, data distribution, number of repeats. For more information see (Lovell and Omori, 2008; Bright et al., 2011; Lovell, 2012).

## 5 Test acceptance criteria

For an experiment to be considered valid, it needs to include:

• Valid PC: The PC used in the comet experiments is valid or acceptable, if the effect is within the range of mean ±2× standard deviation of historical control data for the same cell line.
• Valid NC: The NC is valid if the effect observed is within the range of mean ±2× standard deviation of historical control data for the same cell line.
• Adequate number of cells and concentrations have been analysed.
• The criteria for selection of the highest concentration of the NMs are met.
• Quality control of test system (mycoplasma test) is shown to be negative

## 6 Historical positive and negative controls

For every laboratory using *in vitro* comet assay, it is highly recommended to build historic controls, both negative as well as positive, for each cell type used. Different cell lines may give different % DNA in tail (background damage level) for the NC. It is also important to demonstrate the ability of the laboratory to perform the assay consistently and to show that the cells used have a low background level of DNA damage, so are capable of picking up a positive effect, with reasonably low variability (OECD, 2014b). When reporting results, it is informative to show average and minimum-maximum values of negative/positive historical controls from last 10–20 experiments performed in the laboratory.

**FIGURE 3:**
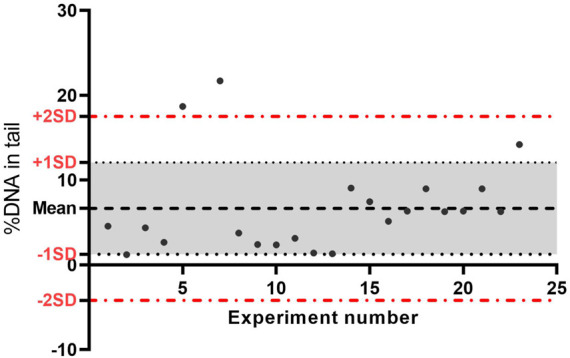
Data quality control chart of negative controls (no enzyme) from 22 experiments performed over time on A549 cells. The central line represents the average of all negative control data, the upper line (red) is for the upper control limit (+2SD), and the lower line (red) is for the lower control limit (−2 SD). The lines are determined from the laboratory historic data. By comparing current data to these lines, we can draw conclusions about whether the negative control is acceptable; if the value is outside the upper line, the negative control is not acceptable. SD, standard deviation.

A laboratory´s historic database for NC and PC data for relevant cell lines needs to be up to date. To define the acceptable range for DNA damage level on NC and PC, controls, calculated Mean ±SD of the data can be used. With the aim of monitoring the proficiency of the *in vitro* assays, both initially and over time, the use of quality control charts to assess the historic control databases is recommended (For more information, see report on statistical issues related to OECD TGs on genotoxicityand Genetic toxicology Guidance documents) (OECD, 2014a, 2015). An example of historical controls from the NC using A549 cells without enzyme treatment from our laboratory is presented in Figure 3.

## 7 Evaluation and interpretation of the results

In addition to fulfilment of the acceptance criteria, since in the case of NMs a concentration response is not always observed (due to agglomeration at higher concentrations), in the EU FP7 project NanoREG2 we developed modified criteria for positive, negative and equivocal genotoxicity response (El Yamani et al., 2022).

A compound is considered positive if there is:

A. a significant increase in strand breaks or oxidised DNA bases at two of the tested concentrations (<30% cytotoxicity) compared to negative controls OR
B. a significant increase in strand breaks or oxidised DNA bases at one of the tested concentrations compared to negative controls AND a concentration response relationship when evaluated with an appropriate trend test.

A compound is considered equivocal if there is a significant concentration response OR a statistically significant increase in strand breaks or oxidised DNA bases at one of the tested concentrations (<30% cytotoxicity) compared to negative controls.

A compound is considered negative if none of the above criteria are met; additionally, all results are inside the distribution of the historical negative controls.

**FIGURE 4:**
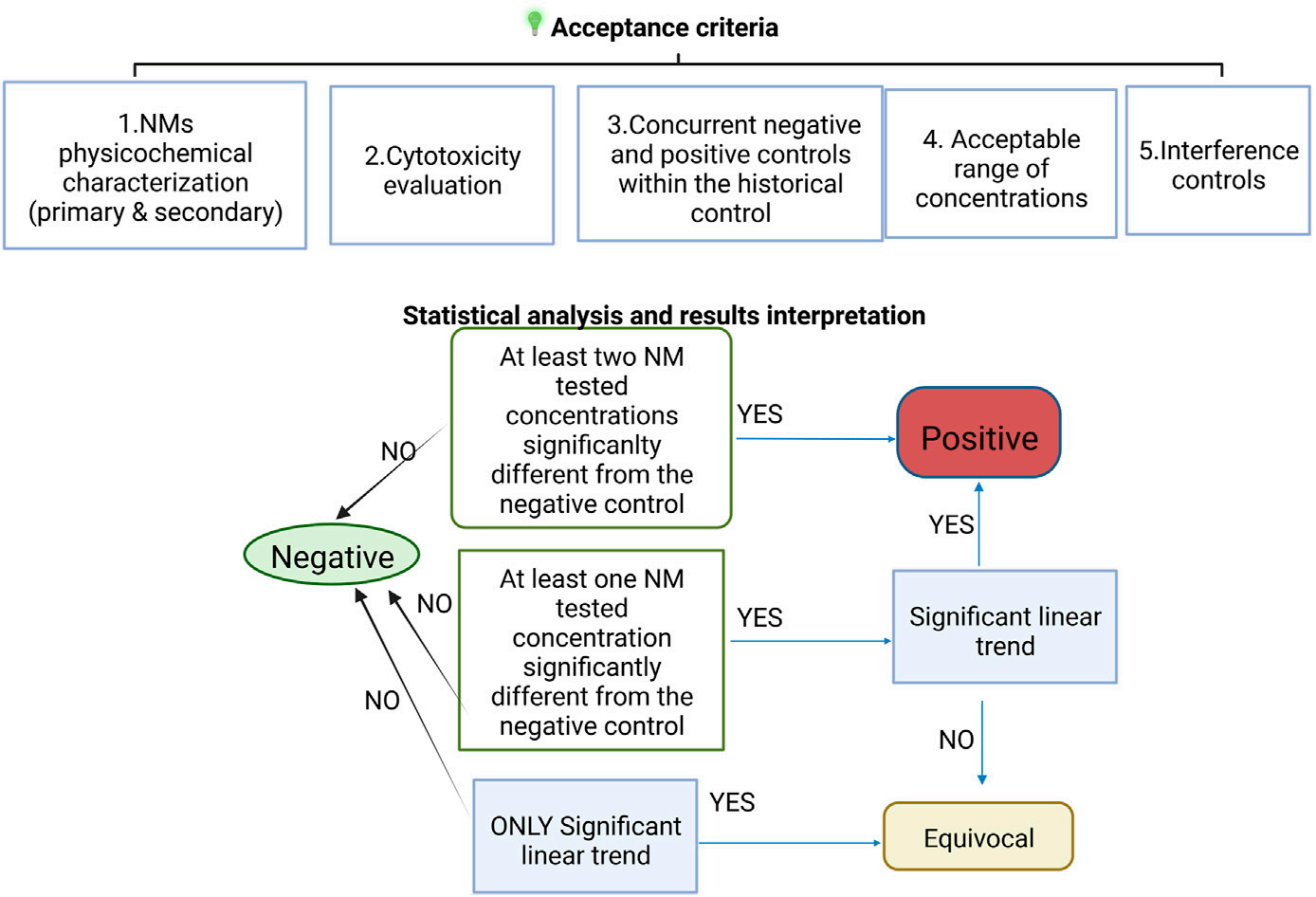
Scheme of the test acceptance criteria for NMs testing including the interpretation of the statistical analysis results.

A scheme summarising the acceptance criteria and evaluation of the NMs effect is shown in Figure 4.

To summarize, negative results indicate that, under the test conditions, the tested NMs does not induce DNA damage in the cultured cells used. Positive results indicate that, under the test conditions, the NM tested is potentially genotoxic *in vitro*. If the response is neither clearly negative nor positive, the test substance is considered equivocal and further testing is needed.

## 8 Interpretation of interference control results

The interpretation of results from interference controls depends on the set-up followed. For instance, if the controls are intended to investigate whether a direct physical interference may occur after cell embedding between residual NMs and DNA, influencing DNA migration, results are compared with those from the controls without NMs. If there is a significant increase or decrease in % DNA in tail compared with the NC or PC, we may conclude that there is an interference. More details on how to perform and interpret interference controls are under preparation for a separate manuscript.

## 9 Discussion

The miniaturized enzyme-linked comet assay is one of the few key assays available to study DNA damage and DNA oxidation induced by NMs. In this paper we described an optimized version of this assay *in vitro* which has been applied in several projects including EU-projects NanoTEST, NanoTOES, NANoReg, NanoREG2 (Magdolenova et al., 2014; Garcia-Rodriguez et al., 2019) and others; it is also being followed within the current projects H2020 RiskGONE and NanoSolveIT, to carry out hazard assessment of several NMs.

The miniaturized version of the comet assay is enormously advantageous for NMs testing, enabling testing of a large number of NMs within the same experiment, minimizing variability and increasing robustness. The miniaturization of the comet assay using 12- gel or even 96 gel format has already been successfully applied to study many NMs (Azqueta and Dusinska, 2015; Di Bucchianico et al., 2017; El Yamani et al., 2017; Garcia-Rodriguez et al., 2019; Collins et al., 2022).

Owing to their physicochemical properties, NMs are more challenging to test for genotoxicity than their counterpart bulk chemicals. The conventional procedure for *in vitro* comet assay testing of chemicals has been adapted to meet the specific needs of NMs testing. Acceptance criteria have been revised, as well as requirements for test validity. The effect of NMs on DNA is highly related to their physicochemical properties both intrinsic (as pristine) and extrinsic (e.g., in medium, vehicle). Characterization of NMs in terms of pristine TEM size, size distribution in cell culture medium before during and after exposure, dissolution rate, zeta potential, and cellular uptake is needed to fully understand the mechanisms and mode of action of NMs (Huk et al., 2015a; Magdolenova et al., 2015). When assessing NMs for their hazards, it is necessary to follow a standard protocol of dispersion and sonication, even including calibration of the sonicator. We previously published a testing strategy to increase the robustness and the throughput of this assay, allowing testing several NMs, different cell lines, different time points and different endpoints within same experiment (Dusinska et al., 2015).

Interpretation of comet assay data when testing NMs may also be challenging. The concentration-response relationship for NMs is not straightforward as it is for chemicals; in other words, an increase in concentration does not necessarily mean an increase in effect. On the contrary, it may lead to an increase of aggregation/agglomeration which will affect the cellular uptake and final effect. The comet assay is a sensitive method and the background level of DNA damage in cells varies. Historical controls are important to demonstrate the technical competence of a given laboratory, and its familiarity with the assay (Hayashi et al., 2011). The OECD has clearly stated how important it is to compare control data in a given experiment with historical controls (negative and positive) for the cell lines to be used (OECD, 2015). The interpretation of genotoxicity results was discussed at the 2009 International Workshop on Genotoxicity Testing (IWGT) in Basel, Switzerland (Hayashi et al., 2011). We stress the importance of historical controls data and encourage users of this assay to build their historical control database as recommended by the OECD (Hayashi et al., 1989; Adler et al., 1998). Providing historical control data, or other proof of validity of the assay, should be a requirement when publishing *in vitro* comet assay data relating to the hazard assessment of any substance, including NMs.

The selection of concentrations of an NMs for genotoxicity testing *in vitro* can only be defined when information on cytotoxicity is available. Exposure to cytotoxic concentrations can lead to false positive genotoxicity results, and so definition of the cut-off for cytotoxicity is important. We also recommend the use of modified criteria for positive, negative and equivocal response for NMs. The responses can be expressed by numbering each category—1-negative, 2-equivocal and 3-positive. This is relevant when integrating physicochemical properties and *in vitro* toxicological data with *in silico* tools as described in El Yamani et al., 2022 and for developing predictive models.

Potential interference of NMs with the testing methods has become a topic of concern already for many years. Most conventional toxicity assays rely on colorimetric/fluorometric principals, and the particular physical properties of NMs mean that they are prone to interfere with testing methods, as shown in several studies (Ong et al., 2014). Possible interference between NMs and the comet assay has been investigated (Kain et al., 2012; Magdolenova et al., 2012; Karlsson et al., 2015; Ferraro et al., 2016; George et al., 2017; Jalili et al., 2022). For instance, Ferraro et al., 2016, showed a possible interaction between naked DNA and NMs just before the electrophoresis step (Ferraro et al., 2016). Other authors questioned the use of comet assay for testing photosensitive NMs (Karlsson et al., 2015). Therefore, we strongly stress the importance of including additional controls to check for possible NMs interference. A thorough approach is being developed further under H2020 RiskGONE; a joint review paper on NMs interference is under preparation.

The comet assay is widely used for testing genotoxicity of chemicals and is the most used method for testing genotoxicity of NMs. It is thus important to follow standard protocol that addresses all challenges related to NMs features. Hence, to develop OECD TG for *in vitro* comet assay is urgently needed.

## Data Availability

The original contributions presented in the study are included in the article/Supplementary Material, further inquiries can be directed to the corresponding author.
